# HERPUD1 suppresses porcine epidemic diarrhea virus replication by recruiting HRD1 to degrade viral ORF3 protein

**DOI:** 10.1128/jvi.00626-26

**Published:** 2026-06-17

**Authors:** Yafei Xu, Chunxiao Mou, Yingjie Xiang, Huili Liu, Wenbin Bao, Zhenhai Chen

**Affiliations:** 1 College of Veterinary Medicine, Yangzhou University 38043, Yangzhou, Jiangsu, China; 2 Jiangsu Interdisciplinary Center for Zoonoses and Biosafety, Yangzhou University 38043, Yangzhou, China; 3 Jiangsu Co-Innovation Center for Prevention and Control of Important Animal Infectious Diseases and Zoonoses, Yangzhou University 38043, Yangzhou, Jiangsu, China; 4 Institute of Animal Husbandry and Veterinary Science, Shanghai Academy of Agricultural Sciences 74594, Shanghai, China; 5 Key Laboratory for Animal Genetics, Breeding, Reproduction and Molecular Design of Jiangsu Province, College of Animal Science and Technology, Yangzhou University 614678, Yangzhou, China; University of Kentucky College of Medicine, Lexington, Kentucky, USA

**Keywords:** PEDV, ORF3, HERPUD1, ER stress

## Abstract

The open reading frame 3 (ORF3) gene of porcine epidemic diarrhea virus (PEDV) is related to virus pathogenicity. A previous study found that PEDV ORF3 protein localizes in the endoplasmic reticulum (ER) to trigger the unfolded protein response (UPR). Nevertheless, the precise interactions and underlying mechanisms between ORF3 and UPR pathway-associated proteins remain largely unexplored. In this study, we demonstrate that ORF3 protein is capable of inducing ER stress through activating the transcription factor-6 (ATF6) pathway and PKR-like ER kinase (PERK) pathway. Through yeast two-hybrid screening, we identified that HERPUD1 interacts with ORF3 protein. Further investigations revealed that HERPUD1 recruits the E3 ubiquitin ligase HRD1 to facilitate ORF3 ubiquitination and subsequent degradation via ER-associated degradation (ERAD). This process in turn alleviates ORF3-induced ER stress and suppresses PEDV replication. Subsequently, it was found that lysine 61 of ORF3 is a crucial site for ORF3 degradation. Then, a recombinant PEDV with ORF3 mutation (rPEDV-ORF3-K61R) was constructed. The rPEDV-ORF3-K61R presented a higher level of ORF3 expression, induced a greater degree of ER stress and more effectively inhibited interferon-β (IFN-β), accompanied by an approximately 0.5 log increase in viral titer compared with the rPEDV. Overall, this study indicates that HERPUD1 inhibits PEDV replication by negatively regulating the stability of PEDV ORF3, providing an updated understanding of the pathogenic mechanism of viral infection.

**IMPORTANCE:**

Porcine epidemic diarrhea virus (PEDV) causes acute diarrhea, vomiting, dehydration, and high mortality in newborn piglets. ORF3 is an important virulence factor of PEDV, which can induce endoplasmic reticulum stress and antagonize interferon production, facilitating virus replication. However, it is unclear whether the host can reversely regulate ORF3 protein and inhibit its functions. This study found that HERPUD1 interacts with PEDV ORF3 protein and mediates ORF3 degradation, thereby inhibiting PEDV replication. The lysine at position 61 of ORF3 protein is a key site for ubiquitination and degradation. Modifying this site can increase the virus titer. This study is the first to identify HERPUD1 as a novel host factor restricting PEDV. These findings provide new insights into the pathogenesis of PEDV and offer new approaches for modifying PEDV vaccine strains to increase virus titer.

## INTRODUCTION

Porcine epidemic diarrhea (PED) is an acute gastroenteric disease caused by porcine epidemic diarrhea virus (PEDV). It is characterized by symptoms such as vomiting, anorexia, watery diarrhea, severe dehydration, and high mortality in neonatal piglets (1). As one of the most prevalent porcine coronaviruses, PEDV has spread globally, resulting in significant economic losses for pig farms. PEDV is an enveloped, positive-sense RNA virus belonging to the family *Coronaviridae*, genus *Alphacoronavirus* (2). The PEDV genome is approximately 28 kb in length and encodes four structural proteins: spike (S), envelope (E), membrane (M), and nucleocapsid (N), in addition to 16 non-structural proteins (NSP1-16) and one accessory protein ORF3 (3).

The sole accessory protein ORF3 of PEDV, located between the S and E genes, encodes a protein of 224 amino acids (4). Research has indicated that PEDV ORF3 gene extends the S-phase, aids in vesicle formation, and enhances the proliferation of PEDV (5). Furthermore, it has been demonstrated that ORF3 interacts with the S protein, suggesting that S and ORF3 may work in concert to regulate PEDV replication *in vivo* (6). Moreover, the ORF3 protein has been shown to inhibit cellular type I interferon (IFN-I) signaling (7). These studies collectively suggest that PEDV ORF3 is involved in various cellular processes. However, the precise mechanisms by which ORF3 participates in host-virus interactions and their impact on the functions of ORF3 remain to be further elucidated.

The endoplasmic reticulum (ER) is a critical organelle in eukaryotic cells, functioning as the primary intracellular reservoir for calcium ions and regulating the synthesis, folding, and modification of proteins (8). Many factors, such as microbial infections, calcium imbalance, and aggregation of unfolded/misfolded proteins in the ER lumen, ultimately lead to ER stress (9). In response to ER stress, the UPR triggers the downstream PERK, inositol-requiring protein-1α (IRE1α), and ATF6 signaling pathways to restore ER homeostasis (10). If a protein fails to reach its functional folded state, it will be retained in the ER lumen where the terminally misfolded proteins will be eliminated via the ER-associated degradation (ERAD) pathway (11). Coronaviruses have been shown to induce ER stress. For instance, PEDV triggers ER stress by activating the PERK, IRE1α, and ATF6 signaling pathways, thereby inducing autophagy to promote viral replication (12). Recent studies have shown that PEDV ORF3 activates the PERK-eIF2α pathways of UPR signaling (13). SARS-CoV-2 infection activates the IRE1α signaling pathway, in which ORF3a and ORF7a are involved in activating ER stress (14, 15). Additionally, SARS-CoV ORF6, ORF8b, and S proteins can also activate ER stress (16, 17).

HERPUD1 is an ER stress-induced protein whose expression is upregulated by the UPR and involved in the degradation of unfolded proteins (18–20). The ER stress-responsive protein ATF6 transcriptionally induces ERAD components, such as HERPUD1, SEL1L, HRD1, and EDEM1 (21). In yeast and mammals, HERPUD1 deletion leads to the accumulation of unfolded proteins and induces persistent ER stress (22, 23). HERPUD1 contains an N-terminal ubiquitin-like (UBL) domain and mediates the degradation of proteins through interactions with the ERAD machinery (24, 25). In this study, we identified HERPUD1 as a novel interacting partner of the PEDV ORF3 protein using a yeast two-hybrid assay. Further analysis demonstrated that HERPUD1 promotes ORF3 degradation, thereby alleviating ORF3-induced ER stress and inhibiting PEDV replication. This study reveals a novel mechanism by which HERPUD1 suppresses PEDV replication through the degradation of the viral ORF3 protein.

## RESULTS

### PEDV ORF3 protein induces UPR through activating PERK and ATF6 pathways

**Fig 1:**
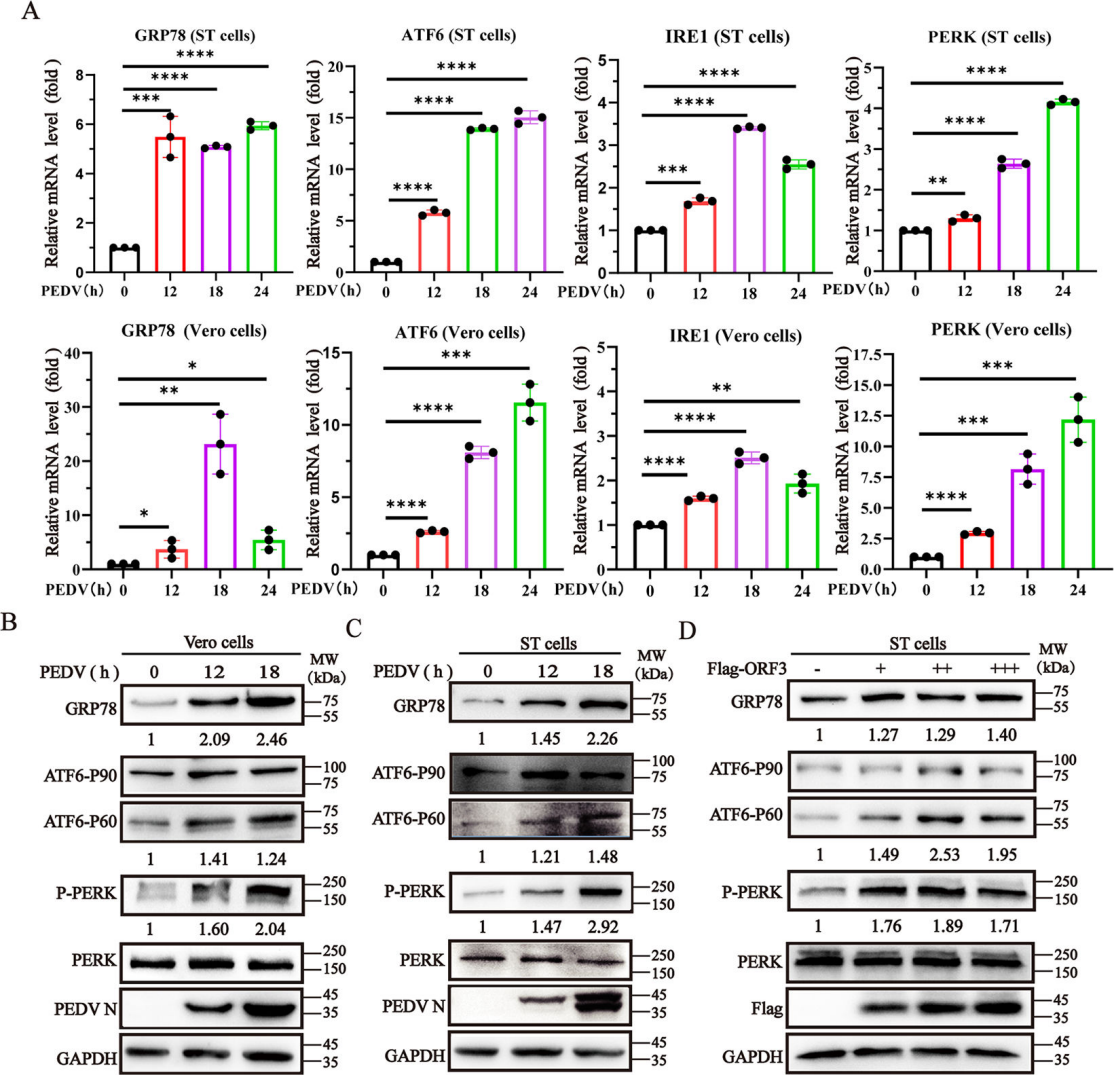
PEDV ORF3 protein induces UPR via the PERK and ATF6 pathways. (**A**) PEDV increased the mRNA levels of ATF6, PERK, and IRE1α. ST cells and Vero cells were infected with PEDV at MOIs of 0.01 and 0.001, respectively, and harvested at the indicated times. The mRNA levels of GRP78, ATF6, PERK, and IRE1 were analyzed by RT-qPCR. (**B and C**) PEDV infection activated the PERK and ATF6 pathways. ST cells and Vero cells were infected with PEDV at MOIs of 0.01 and 0.001, respectively, and harvested at the indicated time points. The expression levels of PEDV-N, p-PERK, PERK, GRP78, ATF6, and GAPDH were detected by Western blot. (**D**) ORF3 induced PERK and ATF6 pathways. ST cells were transfected with increasing doses (0, 0.5, 1, 2 μg) of the Flag-ORF3 for 24 h. The expression levels of p-PERK, PERK, GRP78, ATF6, and GAPDH were detected by Western blot. The experiment was performed three independent times. Relative band intensities were quantified using ImageJ software. Data are shown as mean ± SD (*, *P* < 0.05; **, *P* < 0.01; ***, *P* < 0.001;****, *P* < 0.0001; one-way ANOVA).

In response to ER stress, UPR signaling is activated to minimize ER malfunction. We investigated which of the UPR signaling pathways is activated by PEDV-induced ER stress. Compared with the control group, the mRNA levels of GRP78, ATF6, PERK, and IRE1 were significantly increased after PEDV infection in Vero cells and ST cells (Fig. 1A) (*P* < 0.05). As shown in Fig. 1B and C, compared with the control group, the levels of GRP78, phosphorylation of PERK (p-PERK), and cleaved ATF6 increased significantly in PEDV-infected Vero cells and ST cells. Then, we investigated whether ORF3 could induce the activation of the UPR pathway. The results showed that the levels of cleaved ATF6, p-PERK, and GRP78 in ST cells transfected with pCAGGS-3Flag-ORF3 significantly increased compared with the control group (Fig. 1D). These results indicate that PEDV induces the UPR by activating the PERK, IRE1, and ATF6 pathways, and that ORF3 significantly activates the ATF6 and PERK branches of the UPR.

### HERPUD1 interacts with PEDV ORF3 proteins

**Fig 2:**
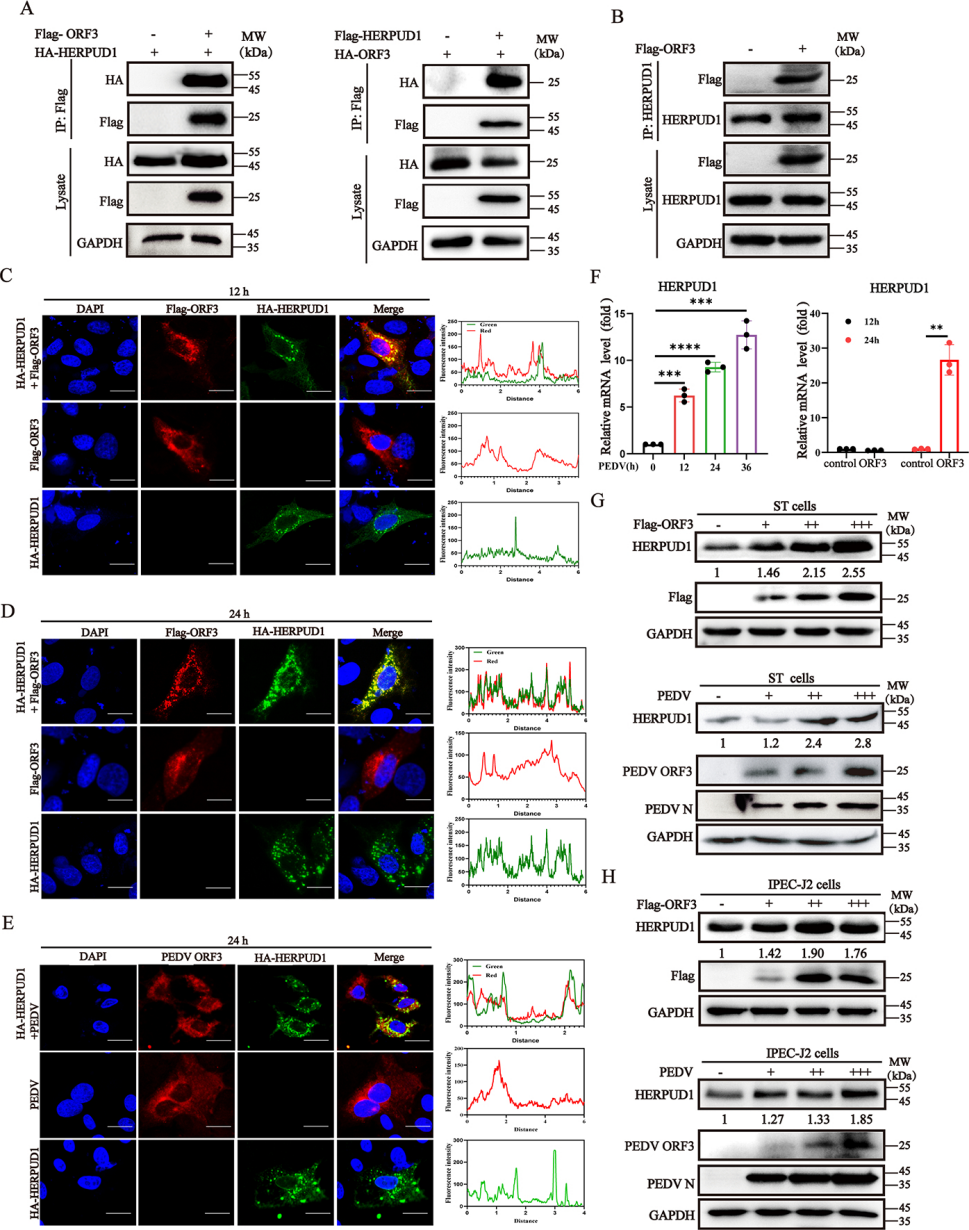
HERPUD1 interacts with the PEDV ORF3 proteins. (**A**) HERPUD1 interacts with ORF3. HEK293T cells were co-transfected with HA-HERPUD1 and Flag-ORF3 or Flag-HERPUD1 and HA-ORF3. Subsequently, co-IP and Western blot were performed using the indicated antibodies. (**B**) HEK293T cells were co-transfected with Flag-ORF3 for 24 h. Co-IP and Western blot were then performed using the indicated antibodies. (**C–E**) HERPUD1 colocalizes with ORF3 in the cytoplasm. Colocalization was performed on the images within the white dashed boxes using IMAGEJ. ST cells were co-transfected with Flag-ORF3 and HA-HERPUD1 for 12 or 24 h. Scale bars, 20 µm (**C and D**). ST cells were transfected with HA-HERPUD1 and later infected with PEDV at an MOI of 0.01. Scale bars, 20 µm. Cells were immunostained with mouse anti-ORF3 and rabbit anti-HA antibodies, followed by DAPI staining, and imaged by laser confocal microscopy (**E**). (**F–H**) ORF3 overexpression and PEDV infection upregulate HERPUD1 expression. ST cells were transfected with Flag-ORF3 or infected with PEDV at an MOI of 0.01. RT-qPCR was performed to analyze the mRNA levels of HERPUD1 (**F**). The expression levels of HERPUD1, PEDV N, and PEDV ORF3 were detected by Western blot in the ST cells (**G**). IPEC-J2 cells were transfected with Flag-ORF3 or infected with PEDV at an MOI of 0.01. The expression levels of HERPUD1, PEDV N, and PEDV ORF3 were detected by Western blot in the IPEC-J2 cells (**H**). The experiment was performed three independent times. Relative band intensities were quantified using ImageJ software. Data are shown as mean ± SD (*, *P* < 0.05; **, *P* < 0.01; ***, *P* < 0.001; ****, *P* < 0.0001; one-way ANOVA).

In this study, we used yeast two-hybrid assays to identify the host protein HERPUD1 as an interaction partner of the PEDV ORF3 protein. This interaction was then confirmed by coimmunoprecipitation (co-IP) (Fig. 2A and B). Furthermore, it is conserved across ORF3 proteins from different viral strains (Fig. S1). Confocal immunofluorescence assays revealed that HERPUD1 colocalized with ORF3 protein in the cytoplasm of ST cells under PEDV infection or ORF3 overexpression (Fig. 2C through E). Subsequently, we examined the impacts of PEDV and ORF3 on the expression of HERPUD1 in ST cells. RT-qPCR and Western blot indicated that HERPUD1 was significantly upregulated in both PEDV-infected and ORF3-overexpressing cells (Fig. 2F and G). We further validated this result in IPEC-J2 cells and in the intestinal tissues of infected piglets (Fig. 2H; Fig. S2). Collectively, these findings demonstrate that ORF3 can induce HERPUD1 expression, and that HERPUD1 interacts with PEDV ORF3.

### Impact of HERPUD1 on the function of ORF3

**Fig 3:**
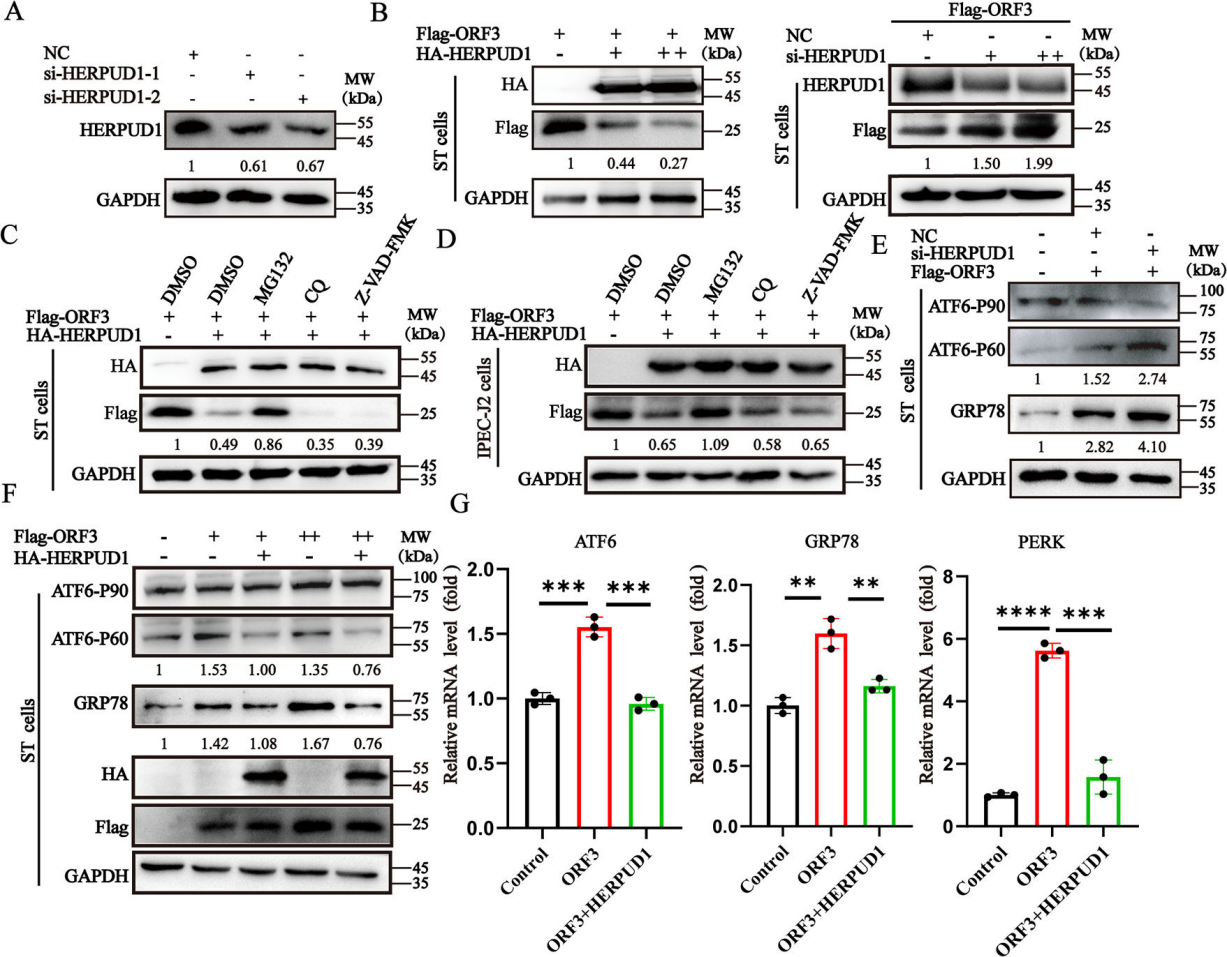
The impact of HERPUD1 on the function of ORF3. (**A**) The interference efficiency of HERPUD1 siRNA. HERPUD1-targeting siRNAs were synthesized and transfected into ST cells. (**B**) HERPUD1 promoted ORF3 degradation in a dose-dependent manner. ST cells were transfected with Flag-ORF3 and increasing amounts of plasmids containing HA-HERPUD1. Cells were collected 24 h after transfection, and the protein level of ORF3 was analyzed by Western blot. The ST cells were transfected with Flag-ORF3 and HERPUD1 siRNA or negative-control siRNA for 24 h. Western blot was used to analyze cell lysates. (**C and D**) Proteasomal inhibitors increased ORF3 expression. ST cells and IPEC-J2 cells were transfected with Flag-ORF3 and HA-HERPUD1 for 24 h. The cells were treated with Z-VAD-FMK (0.5 mM), MG132 (20 μM), and CQ (20 μM). The cell lysates were then analyzed by Western blot. (**E and G**) HERPUD1 negatively regulated ORF3-induced ER stress. ST cells were transfected with Flag-ORF3 and HA-HERPUD1 or siRNA (HERPUD1 siRNA or negative-control siRNA) for 24 h. RT-qPCR analysis of the expression of PERK, ATF6, and GRP78 genes (**E and F**). Western blot analysis of the changes of ATF6 and GRP78 proteins (**G**). The experiment was performed three independent times. Data are shown as mean ± SD (*, *P* < 0.05; **, *P* < 0.01; ***, *P* < 0.001; ****, *P* < 0.0001; one-way ANOVA).

To investigate the impact of HERPUD1 on the function of ORF3, we designed small interfering RNAs (siRNAs) targeting HERPUD1 to knock down the expression of HERPUD1 in ST cells (Fig. 3A). Next, we co-transfected Flag-ORF3 and HA-HERPUD1 plasmids into ST cells. As shown in Fig. 3B, overexpression of HERPUD1 induced the degradation of ORF3 in a dose-dependent manner, while knockdown of HERPUD1 increased the levels of ORF3 protein. The ubiquitin-proteasome and autolysosome pathways are two major intracellular protein degradation pathways in eukaryotic cells (26). To determine the predominant degradation pathway that mediates the degradation of PEDV ORF3 protein by HERPUD1, ST cells and IPEC-J2 cells were co-transfected with Flag-ORF3 and HA-HERPUD1 plasmids and subsequently treated with autophagy-lysosome inhibitors (chloroquine, CQ), ubiquitin-proteasome inhibitor (MG132), and apoptosis inhibitor (Z-VAD-FMK). The results showed that HERPUD1 degrades ORF3 via the proteasome pathway (Fig. 3C and D). We also evaluated the effect of HERPUD1 on ORF3-induced ER stress. Western blot showed that ORF3 increased the levels of cleaved ATF6 and GRP78, while HERPUD1 reduced these protein levels. Furthermore, siRNAs targeting HERPUD1 were transfected into ST cells for 24 h. The expression levels of cleaved ATF6 and GRP78 in the si-HERPUD1 group were significantly higher than in the control group (Fig. 3E and F). To further validate these findings, RT-qPCR results demonstrated that HERPUD1 significantly attenuated the ORF3-induced upregulation of ATF6 and GRP78 mRNA levels (Fig. 3G). Taken together, our results indicate that HERPUD1 promotes the degradation of ORF3 and alleviates ORF3-induced ER stress.

### K61 is the critical ubiquitination site of ORF3

**Fig 4:**
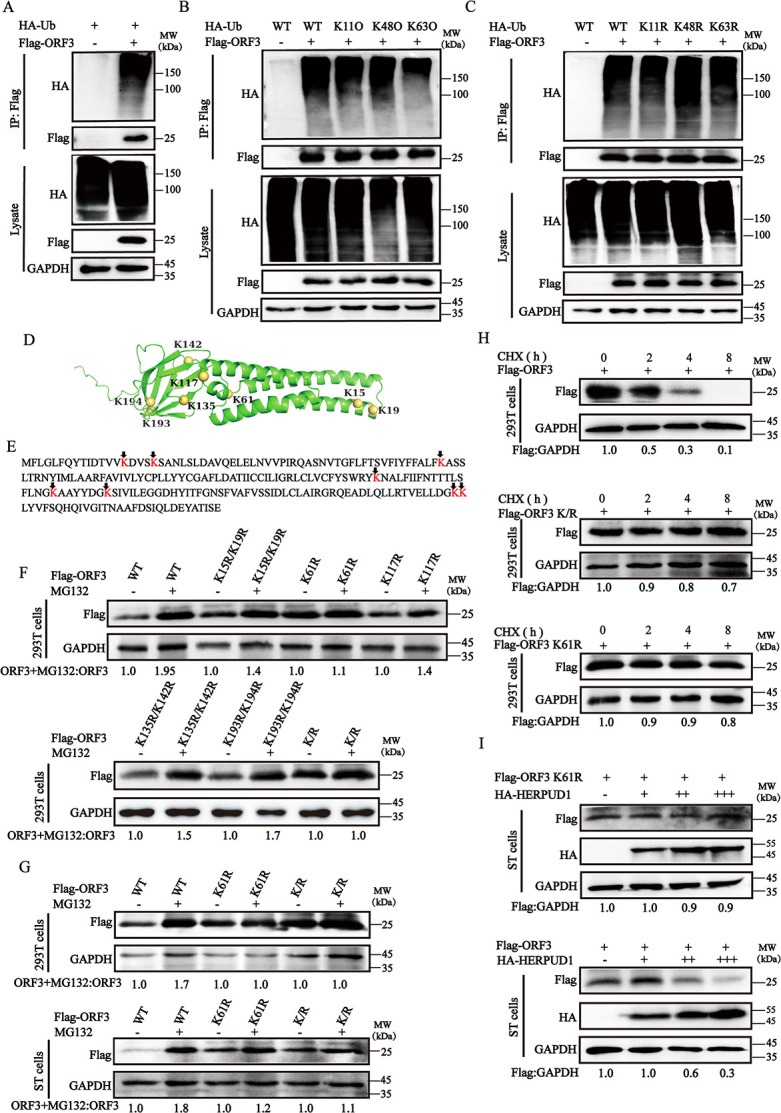
K61 is the critical ubiquitination site of ORF3. (**A**) ORF3 was ubiquitinated. HEK293T cells transfected with Flag-ORF3, HA-Ub, or the empty vector were treated with 20 μM MG132 for 12 h prior to harvest. The interaction between Ub and ORF3 was subjected to pulldown with anti-Flag beads and Western blot with anti-HA antibody to detect the polyubiquitin chains of ORF3. (**B and C**) ORF3 was primarily modified by K11-, K48-, and K63-linked ubiquitin chains in cells. HEK293T cells were transfected with the indicated plasmids for 24 h and then treated with MG132 for 8 h prior to harvest. Cell lysates were subjected to immunoprecipitation with anti-Flag beads, followed by Western blot analysis with an anti-HA antibody to detect polyubiquitinated ORF3. (**D**) The structure of ORF3 protein was visualized by PyMOL with eight lysine residues highlighted in yellow. (**E**) The amino acid sequence of PEDV ORF3 was downloaded from NCBI, and all the lysine residues were marked in red. (**F and G**) Stability of ORF3 lysine mutants. HEK293T cells were transfected with WT-ORF3 and ORF3 mutants (K15R/K19R, K61R, K117R, K135R/K142R, K193R/K194R, and K/R) and then treated with MG132 (20 μM) for 6 h before collection. The protein levels of ORF3 were analyzed by Western blot (**F**). ST cells were transfected with WT-ORF3 and ORF3 mutants (K61R and K/R) and then treated with MG132 (20 μM) for 6 h before collection. The protein levels of ORF3 were analyzed by Western blot (**G**). (**H**) The ORF3-K61R and ORF3-K/R proteins exhibit enhanced protein stability. HEK293T cells were transfected with ORF3-WT, ORF3-K61R, and ORF3-K/R. After 24 h, cells were treated with CHX (100 μg/mL) and collected at the indicated times to detect the protein level of ORF3. (**I**) The ORF3-K61R protein is resistant to HERPUD1-mediated degradation. ST cells overexpressing the ORF3-K61R protein were transfected with increasing amounts of plasmids containing HA-HERPUD1. Cells were collected 24 h after transfection of the HA-HERPUD1 plasmid, and the protein level of ORF3-K61R was analyzed by Western blot. The experiment was performed three independent times. Relative band intensities were quantified using ImageJ software.

If a protein is degraded by the proteasome, it often needs to be ubiquitinated before it can be recognized and hydrolyzed by proteolytic enzymes (27). To further elucidate the mechanism of ORF3 degradation, we sought to examine if ORF3 could be ubiquitinated. The ubiquitination assay showed that ORF3 was conjugated to ubiquitin, forming polyubiquitin chains (Fig. 4A). Subsequently, we investigated the types of polyubiquitin chains linked to ORF3. Ubiquitin can form chains through its own lysine residues and the N-terminal methionine, with K11-, K48-, and K63-linked chains being the most extensively studied. Therefore, we co-expressed ORF3 and either wild-type (WT) ubiquitin (K11O, K48O, K63O) or mutated ubiquitin (K11R, K48R, K63R). The results indicated that ORF3 primarily conjugated with K11-, K48-, and K63-linked ubiquitin chains in cells (Fig. 4B and C).

We further investigated the critical ubiquitination sites of ORF3 involved in proteasome-mediated degradation. ORF3 is a small accessory protein (225 amino acids long) with only eight lysine residues: K15, K19, K61, K117, K135, K142, K193, and K194 (Fig. 4D and E). To determine the ubiquitination sites in ORF3, we constructed lysine mutants, including ORF3-K15R/K19R, ORF3-K61R, ORF3-K117R, ORF3-K135R/K142R, ORF3-K193R/K194R, and the complete lysine-to-arginine mutant ORF3-K/R. We examined the stability of ORF3 mutants with or without MG132 and observed that ORF3-K/R and ORF3-K61R were not degraded (Fig. 4F and G). Following cycloheximide (CHX) treatment, the protein level of ORF3-WT gradually decreased over time. In contrast, the ORF3-K61R and ORF3-K/R mutants remained relatively stable (Fig. 4H). We next overexpressed HERPUD1 in a dose-dependent manner and tested its effect on the ORF3-K61R mutant. The protein levels of ORF3-K61R were not affected by overexpression of HERPUD1 (Fig. 4I). These results confirm that ORF3 is primarily modified by K11-, K48-, and K63-linked ubiquitin chains, and that ORF3 K61 is critical for HERPUD1-mediated regulation of ORF3 stability.

### HERPUD1 recruits HRD1 to regulate the ubiquitin-mediated degradation of ORF3

**Fig 5:**
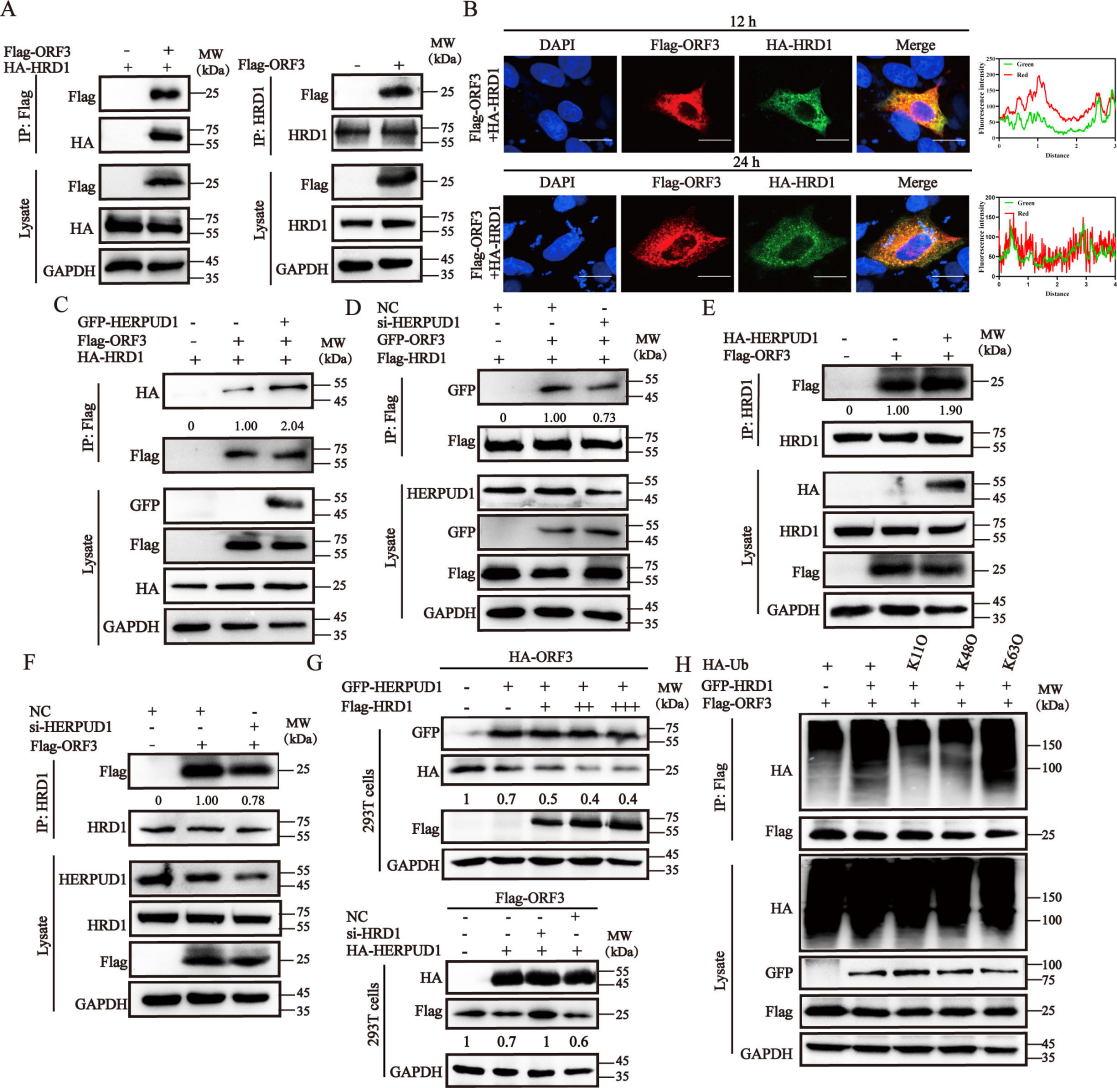
HERPUD1 recruits HRD1 to regulate the ubiquitin-mediated degradation of ORF3. (**A**) HEK293T cells were transfected with HA-HRD1 and Flag-ORF3, or with Flag-ORF3 alone. All cells were treated with 20 μM MG132 for 12 h, and then analyzed by co-IP and Western blot using the indicated antibodies. (**B**) HRD1 colocalizes with ORF3 in the cytoplasm. ST cells were co-transfected with Flag-ORF3 and HA- HRD1 for 12 or 24 h. Cells were immunostained with mouse anti-ORF3 and rabbit anti-HA antibodies, followed by DAPI staining, and imaged by confocal microscopy. Scale bars, 20 µm. Colocalization analysis was performed on the images within the white dashed boxes using IMAGEJ. (**C–F**) HERPUD1 promotes the interaction between ORF3 and HRD1. The cells were analyzed for protein abundance by Western blot. HEK293T cells were transfected for 24 h with HA-HRD1, Flag-ORF3, and GFP-HERPUD1, followed by co-IP with anti-Flag binding beads. Precipitated proteins were analyzed by Western blot (**C**). HEK293T cells were transfected for 24 h with Flag-HRD1, GFP-ORF3, and siRNA (HERPUD1 siRNA or negative-control siRNA), followed by co-IP with anti-Flag beads. Precipitated proteins were analyzed by Western blot (**D**). HEK293T cells were transfected for 24 h with Flag-ORF3 and HA-HERPUD1, followed by co-IP with anti-HRD1 binding beads. Precipitated proteins were analyzed by Western blot (**E**). HEK293T cells were transfected for 24 h with Flag-ORF3 and siRNA (HERPUD1 siRNA or negative-control siRNA), followed by co-IP with anti-HRD1 binding beads. Precipitated proteins were analyzed by Western blot (**F**). (**G**) HRD1 is required for HERPUD1-mediated degradation of ORF3. HEK293T cells were co-transfected with Flag-ORF3, HA-HERPUD1, and either Flag-HRD1 or HRD1-specific siRNA (with non-targeting siRNA as a control). ORF3 protein levels were quantified by Western blot. (**H**) HRD1 modulates K63-linked ubiquitination of ORF3. HEK293T cells were co-transfected with Flag-ORF3, GFP-HRD1, and plasmids encoding HA-tagged WT ubiquitin (HA-Ub-K11O, HA-Ub-K48O, HA-Ub-K63O). Cell lysates were harvested at 24 h post-transfection and subjected to immunoprecipitation with an anti-Flag antibody, followed by Western blot. The experiment was performed three independent times. Relative band intensities were quantified using ImageJ software.

ERAD is a protein quality control system for the degradation of misfolded proteins in the ER, and HRD1 is an important E3 ligase within the ERAD pathway. Therefore, we examined the effect of HRD1 on the degradation of the PEDV ORF3 protein. A co-IP assay showed that ORF3 interacts with HRD1 (Fig. 5A). Furthermore, confocal microscopy demonstrated cytoplasmic colocalization of HRD1 and ORF3 in ST cells (Fig. 5B). As both HERPUD1 and HRD1 are components of the ERAD complex, further exploration is needed to determine whether HRD1 serves as the E3 ligase mediating HERPUD1-induced degradation of ORF3. A subsequent co-IP assay showed that HERPUD1 promoted the interaction between HRD1 and ORF3, and the interaction between HRD1 and ORF3 was weakened after HERPUD1 knockdown (Fig. 5C through F). To further corroborate these findings, HRD1 was knocked down in ST cells using siRNAs. We observed that knockdown of HRD1 signiﬁcantly reversed ORF3 degradation mediated by HERPUD1, while overexpression of HRD1 promoted HERPUD1-induced degradation of ORF3 (Fig. 5G). In addition, HRD1 mediated the K63-linked polyubiquitination of PEDV ORF3 protein (Fig. 5H). The results suggest that HERPUD1 recruits HRD1 to regulate the ubiquitin-mediated degradation of ORF3.

### HERPUD1 inhibits PEDV replication

**Fig 6:**
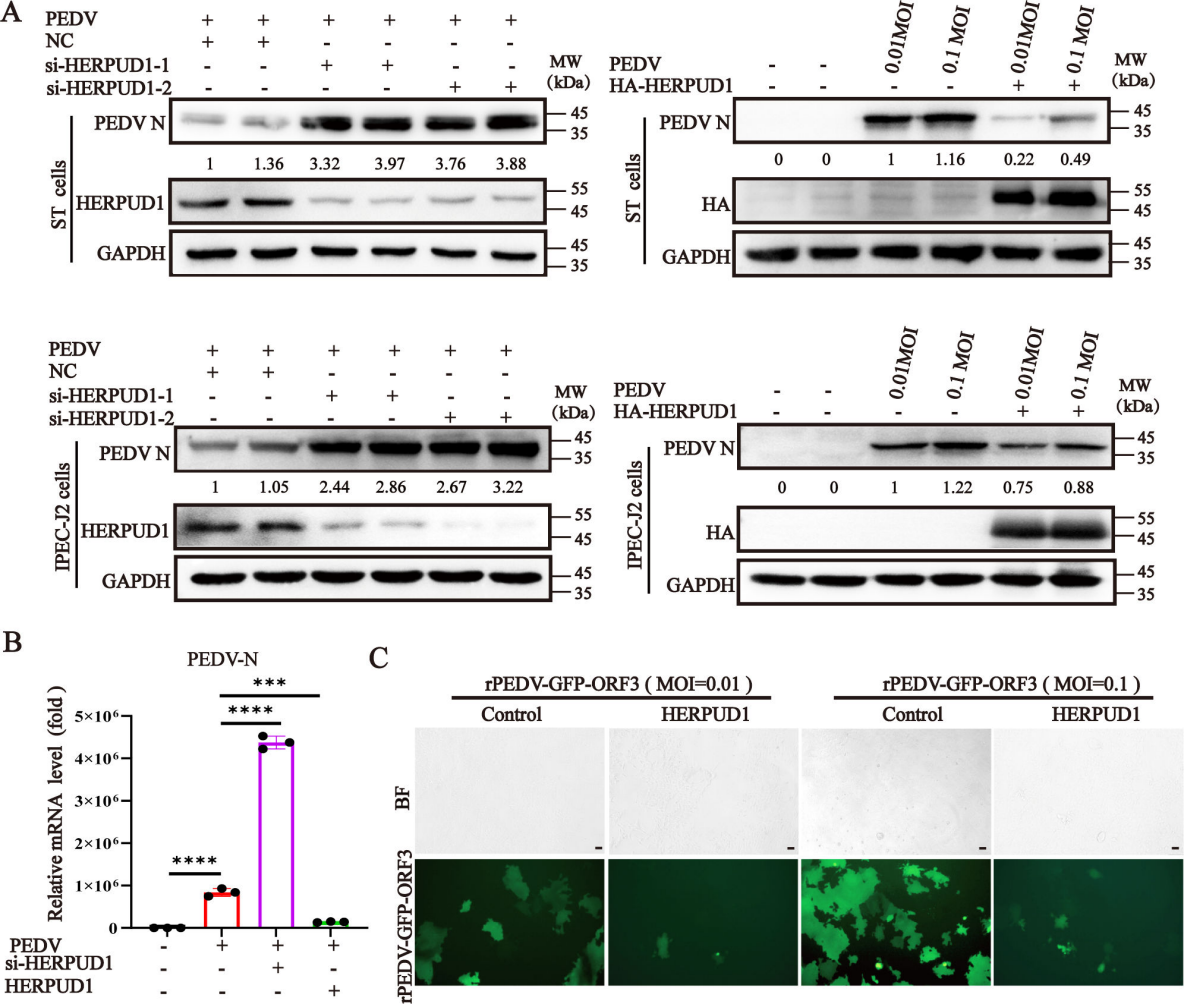
HERPUD1 inhibits PEDV replication (**A**) HERPUD1 inhibits PEDV replication. ST cells and IPEC-J2 cells transfected with either si-HERPUD1 or HA-HERPUD1 for 24 h were infected with PEDV (MOI = 0.01). Western blot was used to quantify N protein. (**B**) ST cells transfected with either si-HERPUD1 or HA-HERPUD1 for 24 h were infected with PEDV (MOI = 0.01). RT-qPCR analysis of the mRNA levels of PEDV-N. (**C**) Effect of HERPUD1 on the replication of the rPEDV-GFP-ORF3 reporter virus was detected by fluorescence microscopy. Scale bars, 20 µm. The brightfield (BF) image shows the overall morphology. The experiment was performed three independent times. Relative band intensities were quantified using ImageJ software. Data are shown as mean ± SD (*, *P* < 0.05; **, *P* < 0.01; ***, *P* < 0.001; ****, *P* < 0.0001; one-way ANOVA).

We further investigated the role of HERPUD1 during PEDV infection. Western blot indicated that overexpression of HERPUD1 significantly suppressed PEDV replication, while the knockdown of HERPUD1 promoted PEDV replication within ST cells and IPEC-J2 cells (Fig. 6A). RT-qPCR further confirmed that overexpression of HERPUD1 significantly downregulated the PEDV N mRNA levels in ST cells at 24 h, whereas silencing HERPUD1 increased the PEDV N mRNA levels (*P* < 0.01) (Fig. 6B). To further validate the role of HERPUD1 in PEDV infection, we overexpressed HERPUD1 and later infected with rPEDV-GFP-ORF3 reporter virus at a multiplicity of infection (MOI) of 0.01. The results demonstrated that HERPUD1 inhibited rPEDV-GFP-ORF3 replication (Fig. 6C). Together, these results indicate that HERPUD1 inhibits PEDV replication.

### rPEDV-ORF3-K61R showed a strong replication ability *in vitro*

**Fig 7:**
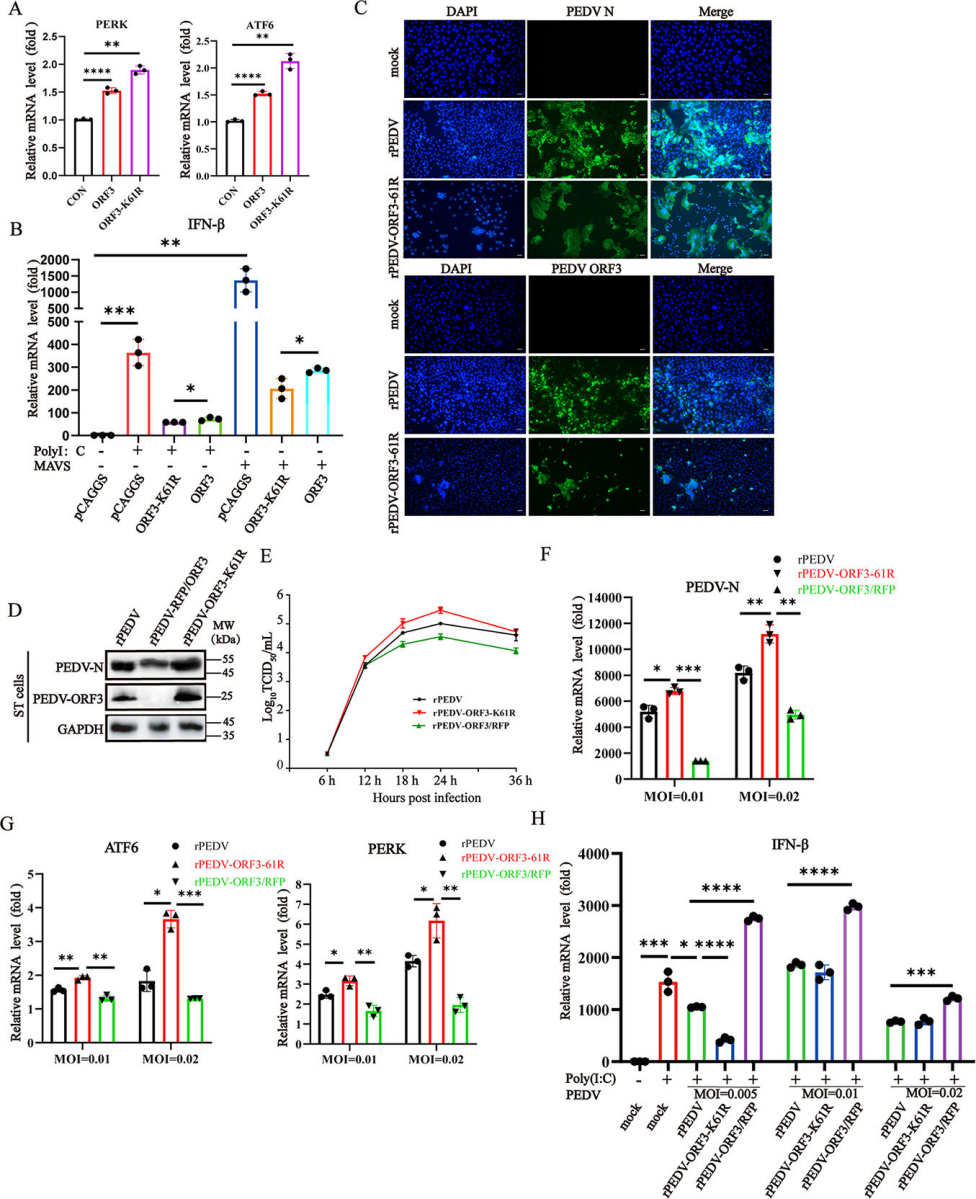
rPEDV-ORF3-K61R showed a strong replication ability *in vitro*. (**A and B**) The K61R mutation enhances the ability of ORF3 to induce ER stress and antagonize IFN-β responses. ST cells were transfected with WT-ORF3 or ORF3-K61R for 24 h. The mRNA levels of PERK, ATF6, and GAPDH were analyzed by RT-qPCR (**A**). ST cells were transfected with WT-ORF3 or ORF3-K61R for 24 h and then transfected with Poly(I:C) (1 μg/mL). The mRNA levels of GAPDH and IFN-β were analyzed by RT-qPCR (**B**). (**C**) IFA analysis of rPEDV- and rPEDV-ORF3-K61R-infected Vero cells at 24 h post-infection, using an anti-PEDV N monoclonal antibody and an anti-PEDV ORF3 monoclonal antibody. Scale bar = 100 µm. (**D**) ST cells were infected with rPEDV, rPEDV-ORF3-K61R, or rPEDV-ORF3/RFP at an MOI of 0.01 for 24 h, and the expression levels of ORF3 and N proteins were quantified by Western blot analysis. (**E**) Supernatants were harvested at the indicated times and titrated for virus titer using the TCID_50_ method. Vero cells were infected with rPEDV, rPEDV-ORF3-K61R, or rPEDV-ORF3/RFP (MOI = 0.001) for 6, 12, 18, 24, and 36 h. (**F**) ST cells were infected with rPEDV, rPEDV-ORF3-K61R, or rPEDV-ORF3/RFP at MOIs of 0.01 or 0.02 for 24 h. The mRNA levels of PEDV-N and GAPDH were analyzed by RT-qPCR. (**G**) ST cells were infected with rPEDV, rPEDV-ORF3-K61R, or rPEDV-ORF3/RFP at MOIs of 0.01 or 0.02 for 24 h. The mRNA levels of PERK, ATF6, and GAPDH were analyzed by RT-qPCR. (**H**) ST cells were infected with rPEDV, rPEDV-ORF3-K61R, or rPEDV-ORF3/RFP. Cells were transfected with Poly(I:C) (1 μg/mL). The mRNA levels of IFN-β were analyzed by RT-qPCR. The experiment was performed three independent times. Data are shown as the means ± SD (*, *P* < 0.05; **, *P* < 0.01; ***, *P* < 0.001; ****, *P* < 0.0001; one-way ANOVA).

PEDV ORF3 has been reported to inhibit IFN-I production and induce ER stress (7, 13). We further examined the effects of the ORF3-K61R on these pathways. Overexpression of ORF3-K61R led to a more significant upregulation of PERK and ATF6 mRNA levels and a more effective suppression of MAVS- and Poly(I:C)-mediated IFN-β induction than WT-ORF3 (Fig. 7A and B) (*P* < 0.05). Together, these findings indicate that the K61R mutation enhances the ability of ORF3 to trigger ER stress and antagonize IFN-β responses. To investigate whether mutation of lysine 61 in ORF3 has an effect on PEDV replication, we generated a recombinant PEDV (named as rPEDV-ORF3-K61R) by replacing ORF3-WT with ORF3-K61R. To further confirm the rPEDV-ORF3-K61R virus, the expression of PEDV N and PEDV ORF3 in rPEDV-ORF3-K61R and rPEDV-infected cells was detected by IFA (Fig. 7C). Western blot analysis showed that rPEDV-ORF3-K61R exhibited a higher level of ORF3 expression (Fig. 7D). TCID_50_ assay indicated that rPEDV-ORF3-K61R virus increased by approximately 0.5 log in viral titer compared with the rPEDV at 24 h (Fig. 7E). Similarly, RT-qPCR analysis revealed a significantly elevated PEDV-N mRNA level in ST cells infected with rPEDV-ORF3-K61R compared with rPEDV at MOIs of 0.01 and 0.02 (Fig. 7F) (*P* < 0.05). Then, we examined the UPR in infected ST cells. Infection with rPEDV-ORF3-K61R resulted in markedly elevated mRNA levels of PERK and ATF6, indicating that the rPEDV-ORF3-K61R induced stronger ER stress (Fig. 7G) (*P* < 0.05). Next, we investigated whether rPEDV, rPEDV-ORF3-K61R, and rPEDV-ORF3/RFP had different abilities to induce IFN-β expression. ST cells were infected with rPEDV, rPEDV-ORF3-K61R, and rPEDV-ORF3/RFP and subsequently transfected with Poly(I:C). RT-qPCR indicated that rPEDV-ORF3-K61R more effectively suppressed Poly(I:C)-mediated induction of IFN-β compared with rPEDV and rPEDV-ORF3/RFP at the MOI of 0.05 (Fig. 7H) (*P* < 0.01). Taken together, these results demonstrated that rPEDV-ORF3-K61R induces stronger ER stress, inhibits IFN-β more effectively, and replicates more efficiently than rPEDV *in vitro*.

## DISCUSSION

Upon coronavirus infection, the virus replicates while simultaneously synthesizing large quantities of viral proteins, leading to the accumulation of unfolded and misfolded proteins. This increases the workload of the ER, generally inducing ER stress and initiating the UPR. These processes trigger a series of signaling cascades that, in turn, affect viral replication (28, 29). Here, we demonstrated that infection with the alphacoronavirus PEDV induced ER stress and triggered the UPR signaling pathway (PERK, IRE1α, and ATF6). It has been reported that the E and ORF3 proteins of PEDV induce ER stress, while ORF3 mediates this response through upregulation of GRP78 and activation of the PERK pathway (13, 30). Consistent with previous reports, we further found that ORF3 can activate the ATF6 pathway. Collectively, these findings indicate that ORF3 induces ER stress and activates the ATF6 and PERK arms of the UPR. Nevertheless, the underlying mechanisms between ORF3 and UPR-associated proteins remain largely unexplored.

To gain deeper insights into the interaction between the ORF3 protein and host proteins, as well as its role in PEDV infection, 25 proteins have been identified as potential ORF3 interacting partners through a yeast two-hybrid assay (Fig. S3). Pathway enrichment analyses of the identified proteins showed that HERPUD1 is involved in the protein processing pathway in the ER. In this study, we confirmed the interaction between HERPUD1 and ORF3 using Co-IP. As a key early effector of ER stress, HERPUD1 orchestrates the export of unfolded or misfolded proteins from the ER to the cytosol and targets their ubiquitinated forms for proteasomal degradation, thereby maintaining protein homeostasis (19, 24). This study explored the regulatory role of HERPUD1 in ORF3. The results indicate that HERPUD1 strongly interacts with ORF3, leading to downregulation of ORF3 and alleviation of ORF3-induced ER stress.

Ubiquitination is a ubiquitous post-translational modification of proteins. The virus and the host play a game in the regulation of ubiquitination: a critical aspect of virus-host interactions. Viruses exploit ubiquitination to evade innate immunity by inducing the degradation or inhibiting the activation of essential host proteins, while hosts utilize ubiquitination to counteract infection by triggering immune responses, targeting viral proteins for degradation, or modulating their functions (31–34). Numerous studies have clearly shown that host cells use the ubiquitination mechanism strategically to precisely regulate viral protein stability. For example, with the participation of E3 ubiquitin ligase HUWE1, the ORF3 protein of MERS-CoV is ubiquitinated, and then it is degraded through the ubiquitin-proteasome pathway, leading to a significant decrease in the ability of ORF3 to induce apoptosis (35). In this study, we found that PEDV ORF3 degradation was reversed via the proteasome inhibitor MG132, and the ORF3 interacts with Ub. These findings collectively suggest that the PEDV ORF3 protein is degraded via the ubiquitin-proteasome pathway. Furthermore, we investigated which of the lysine residues on PEDV ORF3 protein underwent ubiquitination, leading to its degradation. Therefore, we generated a group of ORF3 genes with lysine mutations. The results showed that K61 is the key site for ubiquitination of ORF3. Moreover, the protein level of ORF3-K61R is not affected by HERPUD1, differing from the one of ORF3. These results indicate that the K61 site of ORF3 is critical for HERPUD1-mediated regulation of ORF3 stability.

ERAD functions as a protein quality control system, facilitating the degradation of misfolded proteins within ER. In this process, targeted substrates are ubiquitinated by ER-associated E3 ubiquitin ligases, exemplified by HRD1 (36, 37). Subsequently, ubiquitinated protein substrates are extracted from the cell membrane by VCP/p97 and delivered to the 26S proteasome for degradation (38, 39). The mammalian ubiquitin ligase HRD1 serves as the central component of a complex that facilitates the degradation of misfolded proteins via the ubiquitin-proteasome-dependent process of ERAD. Here, this study explores the specific E3 ligase involved in PEDV ORF3 degradation. We first confirmed the interaction between E3 ligase HRD1 and PEDV ORF3. Importantly, we identified HERPUD1 as a key factor that recruits HRD1 to ORF3, thereby facilitating its ubiquitination and subsequent degradation. Given that K11-, K48-, and K63-linked ubiquitin chains play important roles in regulating protein degradation (40), we sought to determine the specific ubiquitin chain type involved. The results indicate that HRD1 mediates K63-linked ubiquitination of ORF3. This finding is consistent with literature reports that HRD1 promotes K63-linked polyubiquitination, leading to the degradation of the CDV H protein via the ERAD pathway (41). As this study has shown that HERPUD1 is crucial for the degradation of ORF3, it is interesting to know how it affects PEDV replication. We further found that overexpression of HERPUD1 inhibited PEDV replication, while knockdown promoted virus replication. Taken together, HERPUD1 inhibits PEDV replication by recruiting the E3 ligase HRD1, which mediates K63-linked ubiquitination of ORF3 and targets it for degradation via the ERAD pathway.

Growing evidence indicates that mutations of key lysine residues related to viral protein ubiquitination can markedly influence viral virulence. Previous studies showed that in HIV-1, mutations at lysine 26 (K26) of the Vif protein impair its ability to mediate the degradation of the host restriction factor APOBEC3G, thereby significantly reducing viral replication (42). It has also been reported that mutation at lysine 45 (K45) of MERS-CoV ORF3 protein enhances its stability and strengthens its ability to induce apoptosis. In this study, we found that ORF3 K61 is a key residue for ORF3 degradation. Further analysis of ORF3 sequences from both GI group and GII group strains revealed that the lysine at position 61 was conserved (Fig. S4). To investigate the impact of the ORF3-K61 site on PEDV, we constructed a recombinant virus rPEDV-ORF3-K61R using GX4/2021 (GII-a) infectious clone. The results demonstrated that rPEDV-ORF3-K61R increases ORF3 protein stability, triggers robust ER stress, and enhances the suppression of Poly(I:C)-mediated IFN-β production. Compared with rPEDV, rPEDV-ORF3-K61R showed an approximately 0.5-log increase in viral titer. As the central regulatory network through which host cells respond to ER stress, the UPR plays a dual role in PEDV infection. Previous studies have shown that ER stress-mediated autophagy promotes the replication of PEDV. This study found that the PEDV ORF3 can induce ER stress, activating the PERK and ATF6 UPR signaling pathways. In addition, a previous study showed that PEDV ORF3 helps the virus evade the host’s innate immune response by inhibiting type I interferon signaling. Therefore, our findings indicated that rPEDV-ORF3-K61R may counteract HERPUD1-mediated degradation of ORF3, thereby elevating ORF3 protein levels. This, in turn, could trigger robust ER stress and suppress interferon production, ultimately promoting viral replication.

**Fig 8:**
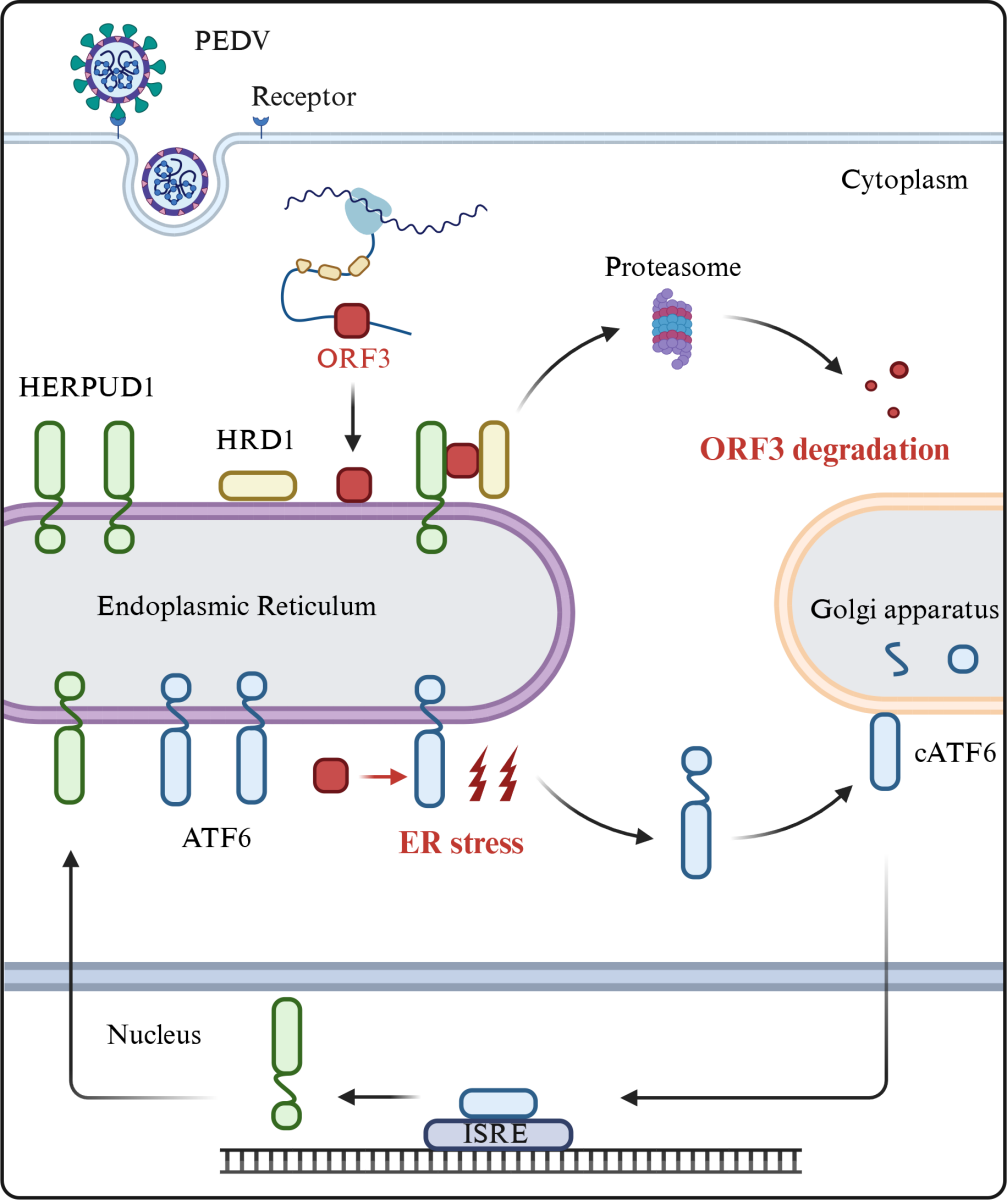
HERPUD1 suppresses PEDV replication by recruiting HRD1 to degrade viral ORF3 protein. PEDV ORF3 protein upregulated expression of HERPUD1 via the ATF6 pathway. Subsequently, HERPUD1 recruits the E3 ubiquitin ligase HRD1 to facilitate ORF3 ubiquitination and subsequent degradation via ERAD, thereby alleviating ORF3-induced ER stress and suppressing PEDV replication.

In summary, the PEDV ORF3 protein induces ER stress, thereby activating the ATF6 pathway and leading to the upregulation of HERPUD1 expression (Fig. 8). Subsequently, HERPUD1 promotes the ubiquitination of ORF3 by recruiting HRD1 and degrades ORF3 through the ERAD pathway, thus alleviating the ER stress caused by ORF3 and inhibiting PEDV replication. Taken together, this study reveals for the first time a new mechanism by which HERPUD1 regulates the degradation of PEDV ORF3 through the ubiquitin-proteasome pathway, thereby inhibiting viral replication. This provides new insights into the pathogenic mechanism of PEDV.

## MATERIALS AND METHODS

### Cells and viruses

Human embryonic kidney (HEK293T) cells, porcine testis (ST) cells, porcine jejunum epithelial (IPEC-J2) cells, and African green monkey kidney epithelial (Vero) cells were cultured in Dulbecco’s modified Eagle medium (DMEM) (Sigma, USA) containing 10% fetal bovine serum (FBS; Lonsera, cat. no. S711-001). The wild-type (WT) PEDV strain GX4/2021 (GIIa PEDV) was propagated in Vero cells using DMEM supplemented with 2 mg/mL trypsin, 0.3% tryptose phosphate broth (TPB) (Sigma, USA), and 1% penicillin-streptomycin. Jejunum tissues from pigs challenged with PEDV strain GX4/2021 were stored in our laboratory (43). Monoclonal antibodies (mAbs) targeting PEDV ORF3 and PEDV N protein were generated in mice. Antibodies against GAPDH (60004-1), PERK (20582-1), GRP78 (66574-1) were purchased from Proteintech. Antibodies targeting DDDDK (AE092), HA (AE105), Myc (AE070), HRD1 (A2605), Phospho-PERK (60004-1), ATF6 (A0202), and EGFP (AE078) were obtained from ABclonal. Antibodies targeting HERPUD1 (26730) were obtained from Cell Signaling Technology. DyLight 488 Goat anti-Mouse IgG and DyLight 549 Goat Anti-Rabbit IgG were acquired from Abbkine Scientific. The PEDV reporter viruses (rPEDV-GFP-ORF3 and rPEDV-RFP/ORF3) used in this study were maintained in our laboratory (44).

### Primer design and plasmids

Specific primers were designed with Primer 5.0 software according to the published porcine GRP78, PERK, HERPUD1, ATF6, IRE1, HRD1, RIG-I, IFN-β, MAVS, TBK1 and GAPDH gene sequences (GenBank accession numbers: XM_021068830.1, XM_003124925.4, XM_021093899.1, XM_021089515.1, XM_013981626.2, XM_003122541.4, XM_021064058.1, NM_001003923.1, NM_001097429.1, NM_001105292.1, and NM_001206359.1). The PEDV ORF3 genes from three different isolates (GenBank accession numbers: OP382083, OP603897, and OQ731917) were each inserted into the pCAGGS-3Flag-2myc vector. Subsequently, the ORF3 gene was cloned into the pEGFP-C3 plasmid, yielding the recombinant plasmid pEGFP-C3-ORF3. The ORF3-K15R/K19R, K61R, K117R, K135R/K142R, K193R/K194R, and K/R were cloned into pCAGGS-3Flag-2myc vector. Genes encoding HERPUD1 and HRD1 were amplified from ST cDNA and inserted into the pcDNA3.1 vector with an N-terminal HA tag. Plasmids expressing Ub-WT, Ub-K11R, Ub-K11O, Ub-K48O, Ub-K48R, Ub-K63O, and Ub-K63R were also stored in our laboratory. All the primers are listed in Tables S1 and S2.

### Quantitative real-time PCR (qRT-PCR)

FreeZol Reagent (R711-01; Vazyme, China) was used to extract total RNA from ST cells and Vero cells, and reverse transcription was performed with HiScript III All-in-one RT SuperMix (R333-01; Vazyme, China). Quantitative PCR (qPCR) was conducted using qPCR SYBR Green Master Mix (11203ES03; Yeasen, China) on a LineGene 9600 Plus system (Bioer, China), and GAPDH was used as the control gene. The fold change in gene expression levels in different treatment groups was calculated using the 2^−ΔΔCT^ method (45).

### Immunofluorescence assay (IFA)

ST cells and Vero cells from different treatment groups were fixed with 4% paraformaldehyde, followed by permeabilization with 0.2% Triton X-100 and blocked using 0.5% BSA. The cells were then incubated with primary antibody at 37°C for 2 h, followed by incubation with secondary antibody (FITC-conjugated goat anti-mouse IgG) and subsequent staining with 4′,6-diamidino-2-phenylindole (DAPI; 2 mg/mL; Solarbio, China) for 5 min. Imaging was performed using a Zeiss LSM 880 NLO confocal laser scanning microscope (Zeiss LSM 880 NLO, Carl, Germany).

### Co-IP

The cells were lysed using lysis buffers (Beyotime Biotechnology, Shanghai, China), followed by collection of the supernatants after centrifugation. The clarified supernatants were subjected to incubation with Flag magnetic beads under low-temperature conditions (4°C) for 6 h. After three sequential washing steps, 5× loading buffer was added to the samples, which were subsequently denatured by heating at 95°C prior to resolution via sulfate-polyacrylamide gel electrophoresis (SDS-PAGE). The electrophoretically separated proteins were transferred onto a polyvinylidene fluoride (PVDF) membrane for subsequent immunoblotting analysis.

### Western blot

The cell proteins were extracted using immunoprecipitation (IP) lysis buffer (Beyotime Biotechnology, Shanghai, China). Subsequently, the total proteins were separated by SDS-PAGE and transferred to PVDF membranes. The membrane was blocked with skim milk and then incubated overnight at 4°C with primary antibodies, followed by incubation with secondary antibodies at 37°C for 1–2 h. Protein bands were detected using an enhanced chemiluminescence (ECL) detection system (Bio-Rad Laboratories, Hercules, CA, USA).

### Yeast two-hybrid assay

The PBT3-STE-ORF3 and porcine alveolar macrophage cDNA plasmids were transformed into the yeast strain NMY51. The transformed yeast was subsequently cultivated, and yeast plasmids were extracted from 32 yeast-positive clones. These plasmids were then transformed into competent bacterial cells and grown on LB (Amp+) plates. Colonies were picked, and bacterial plasmids were amplified. Finally, 25 candidate genes were identified via yeast two-hybrid screening.

### RNA interference

Two siRNAs targeting HERPUD1 and HRD1 were designed by Genecreate (Wuhan, China). The target sequences were as follows: siHRD1-1 (sense: 5′- UGGGCAAGGUGAUGGGCAA-3′), siHRD1-2 (sense: 5′- CUACAGAGCCUGCGCAACA-3′), siHERPUD1-1(sense: 5′- AAGCUAGCACAAAGGUUGCUG-3′), and siHERPUD1-2 (sense: 5′- GCAGUACUACAUGCAGUAU-3′). The siHRD1, siHERPUD1, and negative control siRNA (siNC) were transfected into ST using Lipofectamine RNAiMAX reagent (Thermo Fisher Scientific, USA).

### Generation of recombinant virus using the Red recombination system

The full-length PEDV infectious clone plasmid containing ORF3 K61R (mutation of lysine-61 of ORF3 protein to arginine) was produced by Red recombination using the *E. coli* strain GS1783 (44, 46). The recombinant plasmid was transfected into Vero cells to rescue the rPEDV-ORF3-K61R. Subsequently, rPEDV-ORF3-K61R was used to infect Vero cells, and the viral titer was determined by 50% tissue culture infectious dose (TCID₅₀).

### Viral growth curves

Vero cells were seeded into a 24-well culture plate at a density of 1 × 10⁵ cells per well. The cells were subsequently inoculated with viruses at a multiplicity of infection (MOI) of 0.001. Following a 2-h incubation, the cell culture supernatants were aspirated. Subsequently, the cells were washed twice with PBS, and a maintenance medium was added to the cell monolayers. The cell culture supernatants were collected at different time points. The virus titers were quantified by TCID_50_ assay following the Karber method. The viral growth curves were made by using GraphPad Prism software (version 8.0).

### Statistical analysis

The experimental groups were compared with the mock group using one-way ANOVA in GraphPad Prism (version 8.0) software. The levels of statistical signiﬁcance were deﬁned as follows: *, *P* < 0.05; **, *P* < 0.01; ***, *P* < 0.001; ****, *P* < 0.0001; ns, not significant.

## Data Availability

All data are available from the corresponding author upon reasonable request.

## SUPPLEMENTAL MATERIAL

Fig. S1 jvi.00626-26-s0001.tif HERPUD1 interacts with the PEDV ORF3 proteins, and this interaction is conserved across different viral strains.

Fig. S2 jvi.00626-26-s0002.tif GRP78 and HERPUD1 were significantly upregulated in the intestinal tissues of infected piglets.

Fig. S3 jvi.00626-26-s0003.tif Yeast two-hybrid screening identified 25 candidate host proteins that potentially interact with the PEDV ORF3 protein.

Fig. S4 jvi.00626-26-s0004.tif ORF3 sequences from both GI group and GII group strains revealed that the lysine at position 61 was conserved.

Supplemental legends jvi.00626-26-s0005.docx Descriptive legends for Fig. S1 to S4.

Table S1 jvi.00626-26-s0006.docx Primers used for real-time PCR.

Table S2 jvi.00626-26-s0007.docx Primers used to construct ORF3 mutants.

ASM does not own the copyrights to Supplemental Material that may be linked to, or accessed through, an article. The authors have granted ASM a non-exclusive, world-wide license to publish the Supplemental Material files. Please contact the corresponding author directly for reuse.
