# Supplementary figures and images for: HERPUD1 suppresses porcine epidemic diarrhea virus replication by recruiting HRD1 to degrade viral ORF3 protein
Source: J Virol. 2026 Jun 17;100(7):e00626-26. doi: 10.1128/jvi.00626-26 (PMC13386943; doi:10.1128/jvi.00626-26)

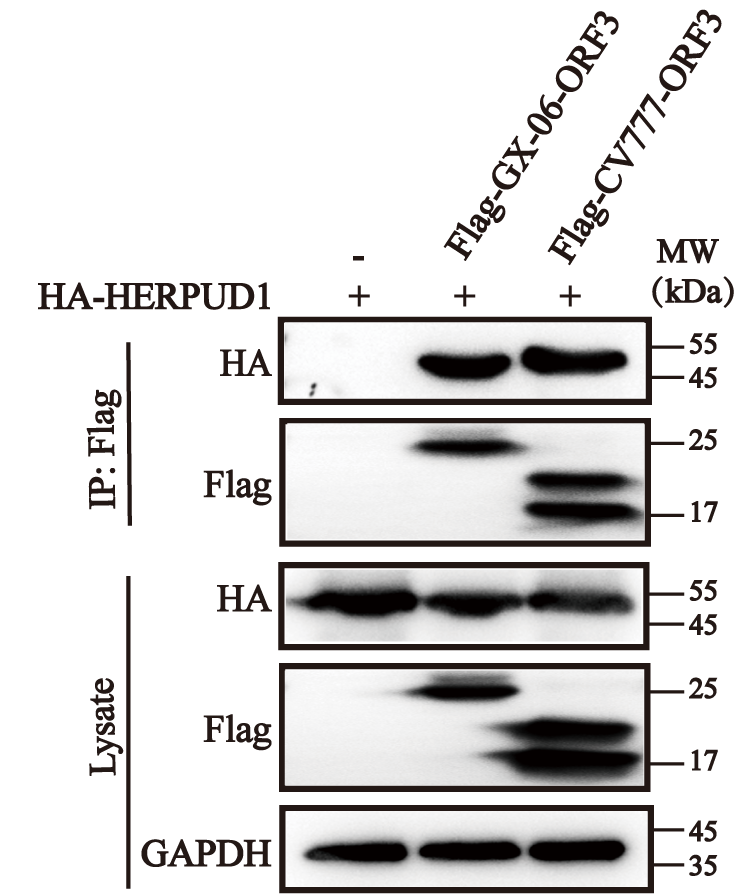

Supplement: Fig. S1 — HERPUD1 interacts with the PEDV ORF3 proteins, and this interaction is conserved across different viral strains. [file jvi.00626-26-s0001.tif]

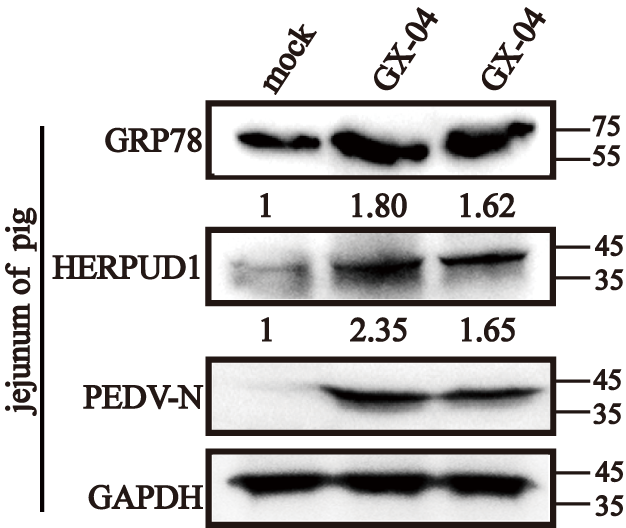

Supplement: Fig. S2 — GRP78 and HERPUD1 were significantly upregulated in the intestinal tissues of infected piglets. [file jvi.00626-26-s0002.tif]

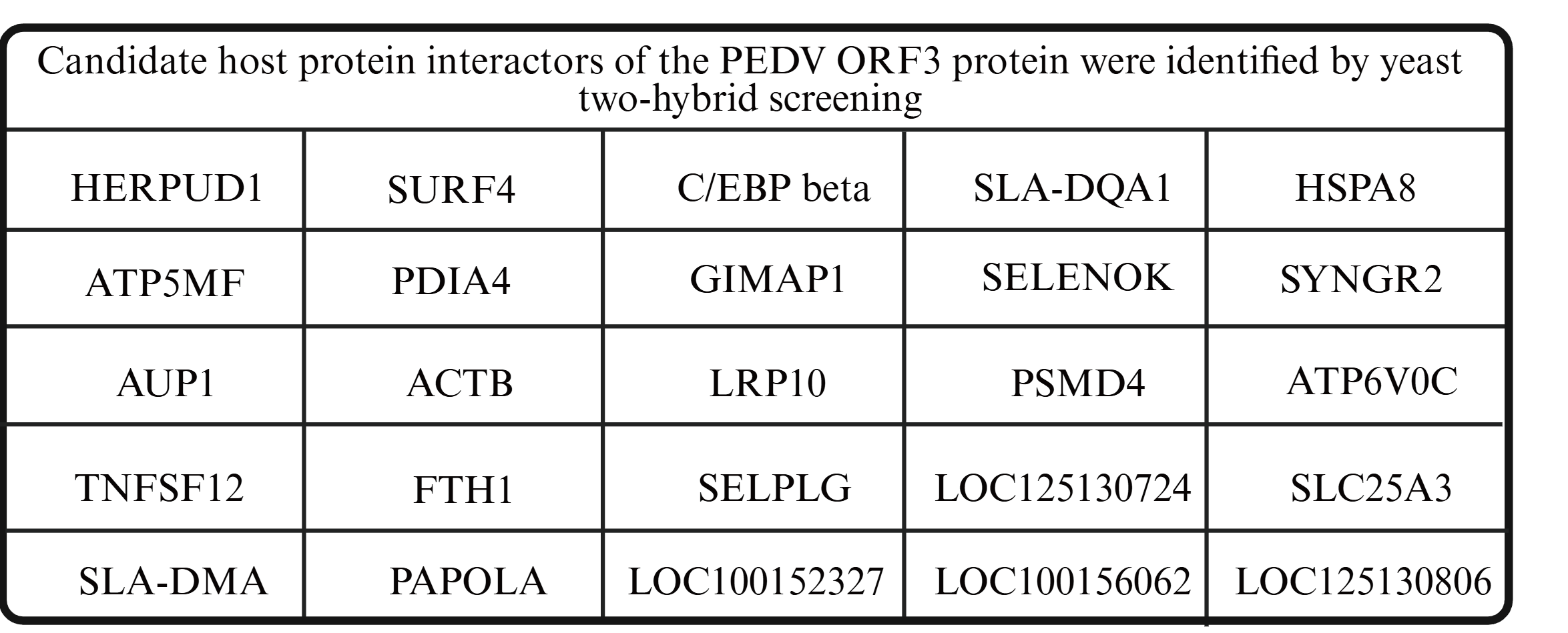

Supplement: Fig. S3 — Yeast two-hybrid screening identified 25 candidate host proteins that potentially interact with the PEDV ORF3 protein. [file jvi.00626-26-s0003.tif]

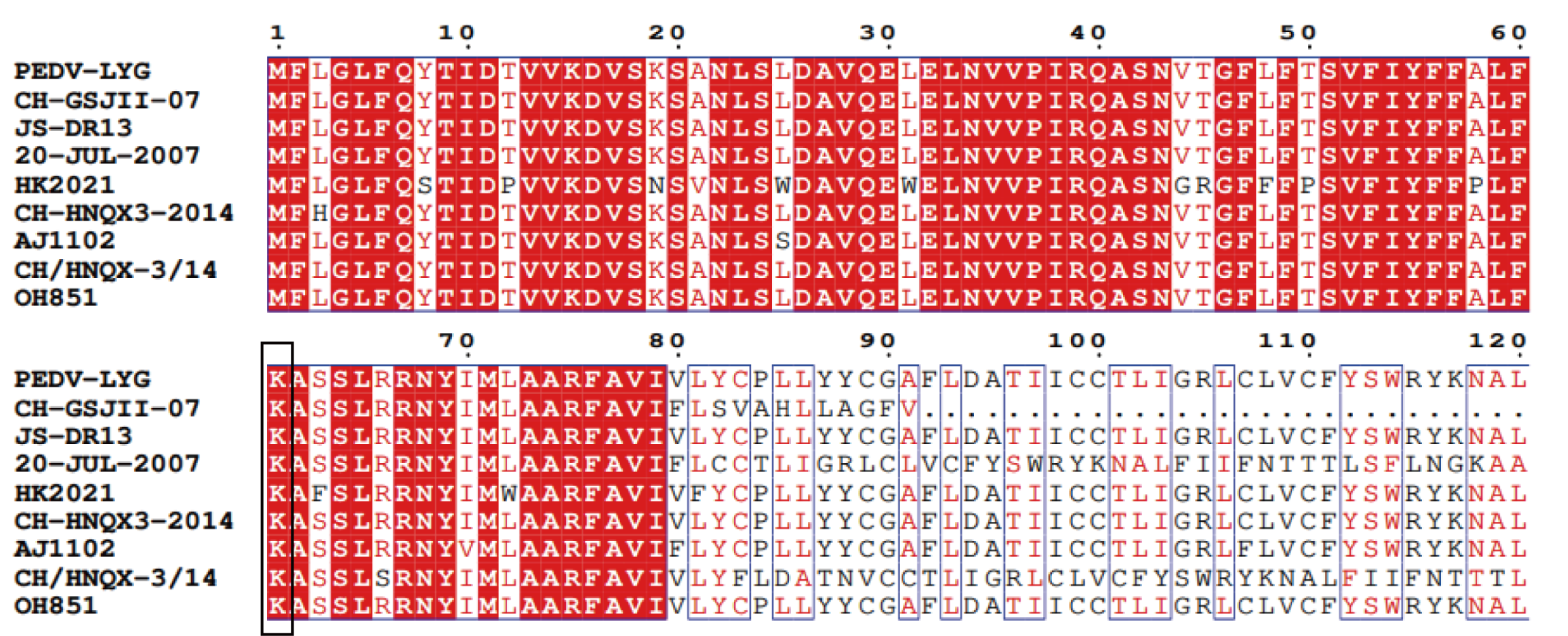

Supplement: Fig. S4 — ORF3 sequences from both GI group and GII group strains revealed that the lysine at position 61 was conserved. [file jvi.00626-26-s0004.tif]
