# Supplementary material for: HERPUD1 suppresses porcine epidemic diarrhea virus replication by recruiting HRD1 to degrade viral ORF3 protein
Source: J Virol. 2026 Jun 17;100(7):e00626-26. doi: 10.1128/jvi.00626-26 (PMC13386943; doi:10.1128/jvi.00626-26)
Supplement: Supplemental legends — Descriptive legends for Fig. S1 to S4. [file jvi.00626-26-s0005.docx]

**Figure legends**

**Fig. S1. HERPUD1 interacts with the PEDV ORF3 proteins, and this interaction is conserved across different viral strains.** HEK-293T cells were co-transfected with HA-HERPUD1 and Flag-GX-06-ORF3 or HA-HERPUD1 and Flag-CV777-ORF3. Subsequently, co-IP and Western blot were performed using the indicated antibodies. All experiments were performed independently with each antibody at least three times.

**Fig. S2. GRP78 and HERPUD1 were significantly upregulated in the intestinal tissues of infected piglets.** Jejunum tissues were collected from 11-day-old piglets that had been challenged orally with 2 mL of PEDV strain GX4/2021 at a dose of 10⁷ TCID₅₀. Piglets in the negative control group received an equal volume of DMEM medium orally. The expression levels of HERPUD1, PEDV N, GRP78 and GAPDH were detected in the jejunum tissues by Western blot. All experiments were performed independently with each antibody at least three times

**Fig. S3. Yeast two-hybrid screening identified 25 candidate host proteins that potentially interact with the PEDV ORF3 protein.**

**Fig. S4. ORF3 sequences from both GI group and GII group strains revealed that the lysine at position 61 was conserved.**
