# Supplementary material for: HERPUD1 suppresses porcine epidemic diarrhea virus replication by recruiting HRD1 to degrade viral ORF3 protein
Source: J Virol. 2026 Jun 17;100(7):e00626-26. doi: 10.1128/jvi.00626-26 (PMC13386943; doi:10.1128/jvi.00626-26)
Supplement: Table S1 — Primers used for real-time PCR. [file jvi.00626-26-s0006.docx]

**Table S1. Primers used for real-time PCR.**

| Primer | Sequence (5′-3′) |
| --- | --- |
| p-GRP78-F | AATGGCCGTGTGGAGATCA |
| p-GRP78-R | GAGCTGGTTCTTGGCTGCAT |
| p-PERK-F | ACTACAAGCGGGAAAGGAGC |
| p-PERK-R | CACCAGTGCAAAAGGAGCAC |
| p-ATF6-F | TCACCGGAGTCCTGGATGAT |
| p-ATF6-F | GAAAGGACAGGGTCGCTTCA |
| p-IRE1-F | TCTTGGGCGGACAGAGTACA |
| p-IRE1-R | CACGGGGGAGGCATAGTTTT |
| PEDV-N-F | GAGGGTGTTTTCTGGGTTG |
| PEDV-N-R | CGTGAAGTAGGAGGTGTGTTAG |
| p-GAPDH-F | TCATCATCTCTGCCCCTTCT |
| p-GAPDH-R | GTCATGAGTCCCTCCACGAT |
| p-HERPUD1-F | CGGGGTACCATGGAGCCGGAACCCGAG |
| p-HERPUD1-R | CGGCTCGAGTCAGTTTGCTATGGCTGGGG |
| p-HRD1-F | GGGGTACCATGTTCCGCACCGC |
| p-HRD1-R | CCGCTCGAGTCAGTGGGCAACCGG |
| p-IFN-β-F | GACATCAAGGAGAAGCTGTGC |
| p-IFN-β-R | TGAAGGTAGTTTCGTGGATGC |
| h-IFN-β-F | TGGGAGGCTTGAATACTGCCTCAA |
| h-IFN-β-R | TCCTTGGCCTTCAGGTAATGCAGA |
| h-GAPDH-F | TCATGACCACAGTCCATGCC |
| h-GAPDH-R | GGATGACCTTGCCCACAGCC |
