# Supplementary material for: HERPUD1 suppresses porcine epidemic diarrhea virus replication by recruiting HRD1 to degrade viral ORF3 protein
Source: J Virol. 2026 Jun 17;100(7):e00626-26. doi: 10.1128/jvi.00626-26 (PMC13386943; doi:10.1128/jvi.00626-26)
Supplement: Table S2 — Primers used to construct ORF3 mutants. [file jvi.00626-26-s0007.docx]

**Table S2. Primers used to construct ORF3 mutants.**

| Primer | Sequence (5′-3′) |
| --- | --- |
| ORF3-K15R/K19R -F | CAATACACGATTGACACAGTTGTCAGAGATGTCTCAAGGTC |
| ORF3-K15R/K19R -R | TCAGAGATGTCTCAAGGTCTGCTAACTTGTCTTTGGATGC |
| ORF3-K61R-F | CTTCTTTGCACTGTTTAGAGCGTCTTCTTTGACGCGC |
| ORF3-K61R-R | CGCTCTAAACAGTGCAAAGAAGTAGATAAAAAC |
| ORF3-K117F | CTCCTGGCGCTATAGAAATGCGCTC |
| ORF3-K117R | GAGCGCATTTCTATAGCGCCAGGAG |
| ORF3-K135R/K142R-F | TGGTAGAGCAGCTTATTATGACGGCAGATCCATT |
| ORF3-K135R/K142R-R | GGATCTGCCGTCATAATAAGCTGCTCTACC |
| ORF3-K193R/K194R-F | GAGCTTCTTGATGGCAGGAGGCTTTATGT |
| ORF3-K193R/K194R-R | AGCCTCCTGCCATCAAGAAGCTCAACAGTTCG |
| ORF3-F | ATGTTTCTTGGACTTTTTCAATACACGATTG |
| ORF3-R | TCATTCACTAATTGTAGCATACTCGTCTAGTTG |
| Overlap-F | ATGACGATGACAAGGGTACCATGTTTCTTGGACTTTTTC  AATACACGATTGAC |
| Overlap-R | GTTTTTGTTCCTCGAGTCATTCACTAATTGTAGCATACTC  GTCTAGTTGAATAGAG |
